# Paediatric diabetes care during the COVID‐19 pandemic: Lessons learned in scaling up telemedicine services

**DOI:** 10.1002/edm2.202

**Published:** 2020-11-21

**Authors:** Christine A. March, Amanda Flint, Diana DeArment, Amy Gilliland, Karen Kelly, Ernesto Rizzitano, Aaron Chrisman, Radhika H. Muzumdar, Ingrid M. Libman

**Affiliations:** ^1^ Division of Pediatric Endocrinology, Metabolism, and Diabetes UPMC Children's Hospital of Pittsburgh Pittsburgh PA USA

**Keywords:** COVID‐19, telemedicine, type 1 diabetes

Paediatric diabetes care relies upon a multidisciplinary approach for the education of and collaborative medical decision‐making with patients and families. It involves healthcare providers, diabetes care and education specialists (formerly known as certified diabetes educators), registered dieticians, medical assistants, nurses, social workers and behavioural health specialists. Behind the scenes, administrative assistants and schedulers also contribute to making this care possible.

Across the world, the COVID‐19 pandemic has upended routine outpatient care, placing new demands on healthcare systems to meet the needs of their patients. Many facilities, including our own, rapidly expanded telemedicine services to facilitate patient care.^1^ Though telemedicine has been utilized for both paediatric and adult diabetes care before,^2^, ^3^, ^4^, ^5^, ^6^, ^7^, ^8^, ^9^, ^10^, ^11^ current endeavours by many institutions are now on an unprecedented scale. In some cases, health systems may have limited existing infrastructure due to previous state law limiting telemedicine services, such as in Pennsylvania.

Prior to COVID‐19, our large, academic centre had minimal telemedicine services for subspecialty care, including diabetes. We present lessons from our experience embarking in telemedicine for routine diabetes appointments and outpatient education. These lessons reflect the feedback received by all members of the division. Physicians, advanced practice providers (APPs), fellows, certified diabetes care and education specialists, nurses and administrative staff provided their viewpoints at their routine meetings to their co‐ordinators/managers who in turn brought back this input to the division leadership. Using this approach, our centre was able to rapidly and successfully scale up our telemedicine services in order to continue providing care to our patients (Figure 1).

**Figure 1:**
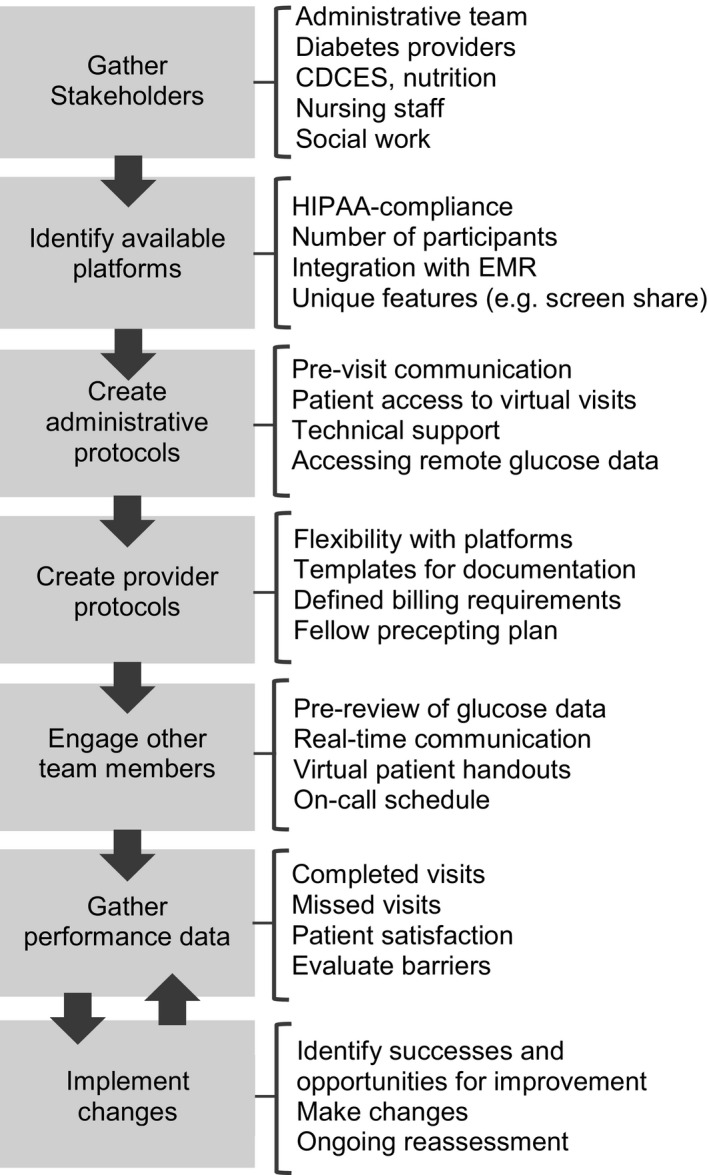
Diagram outlining the procedures to scale telemedicine services during the COVID‐19 pandemic with specific considerations highlighted

Our diabetes centre follows ~2200 patients, (~55% females, ~88% White, 35% with public, 10% with private and 55% with public and private insurance). Prior to COVID‐19, our typical weekly schedule included approximately 200 diabetes appointments, with an average of 80% completed visits (all in‐person). In our first week of telemedicine, we completed 76 of 157 (48%) scheduled visits virtually; by the third week, this increased to 126 of 166 (83%) visits. Since that time, we continued to have more than 80% of ~200 scheduled diabetes visits virtually, reaching almost 90% by the fifth week (Table 1). In some cases, offering virtual visits during this time may have improved our typical no show/cancellation rate by increasing communication with the family in advance of the visit and removing possible barriers to families from inclement weather, transportation or work/education obligations. We summarize key points in Table 2.

**Table 1:** Progression of in‐person to virtual visits during COVID‐19 pandemic

Week start	Scheduled visits	Completed visits	Cancelled visits	No show visits	In‐Person visits	Virtual visits
3/2/20	229	166 (72)	45 (20)	18 (8)	166 (73)	0 (0)
3/9/20	227	169 (74)	37 (16)	21 (10)	169 (74)	0 (0)
3/16/20	190	111 (58)	51 (27)	28 (15)	111 (58)	0 (0)
3/23/20	157	113 (72)	25 (16)	19 (12)	37 (24)	76 (48)
3/30/20	162	119 (73)	35 (22)	8 (5)	0 (0)	119 (73)
4/6/20	153	126 (83)	16 (10)	11 (7)	0 (0)	126 (83)
4/13/20	197	170 (86)	11 (6)	16 (8)	0 (0)	170 (86)
4/20/20	190	169 (89)	5 (3)	15 (8)	1 (<1)	169 (89)

Note

Table displays the number of scheduled return diabetes visits across our main, satellite and outreach locations. Data are presented as n (%). Weeks in early March included as a reference for a typical week. Definitions: completed visits: patients seen either in‐person or virtually; cancelled visits: patient cancelled visit more than 24 hours in advance; no show visits: patient did not arrive for in‐person appointment or could not be reached on the day of appointment for telemedicine.

**Table 2:** Key points summarizing our experience

What we have learned
Decision‐making team should be composed of representatives from all clinical roles
Open lines of communication between administration and providers are essential for rapid sharing of information
Having multiple available telehealth systems can prevent slowdowns in clinic if technical issues arise
Building templates for documentation and billing can enhance provider efficiency
Multidisciplinary telemedicine care with trainees and ancillary providers is possible and essential
Opportunities for improvement
Establish patient‐centred systems to share glucose data with our clinic regardless of device
Tailoring of virtual platforms to suit clinical needs (eg screen sharing)
Creating workarounds to enable completion of needed examinations or laboratory studies
System‐wide administrative processes to facilitate patient enrolment into the EMR portal and streamline communication
Developing an evidence‐based approach to target telemedicine services to certain populations moving forward with formalized patient feedback
Continue to understand how issues with state licensure as well as telemedicine coverage will be addressed in a long‐term care model.

## 1 LESSON 1: TAKE A TEAM APPROACH

Our division includes 17 faculty, eight fellows, 13 APPs, 11 diabetes care and education specialists, four dieticians and two social workers. These providers are supported by a large team of nurses, medical assistants and administrative staff. Rapidly scaling telemedicine services required a joint effort among all personnel and support from leadership to ensure seamless continuity of care. The initial planning phase involved identifying a representative among each of these groups. As we created a framework for transitioning to telemedicine, each of these stakeholders had a role within a series of planning and feedback meetings. This facilitated clear avenues for communication both among and between different groups, allowing for continued reassessment and collective problem solving of emerging issues. As a result, we were able to cohesively develop a uniform approach to telemedicine services in a very short period of time.

It is important to note that no adjustments in the schedules were made when converting from in‐person to virtual visits. The same appointments were kept. The administrative staff contacted patients and families the week ahead of the visits to let families know what to expect, instruct them on the process and give them thorough instructions on how to participate in the portal‐based virtual visit. Instructions on how to join the portal were also provided by emailing them directly to families and posting on the hospital website. Patients that could not be reached initially were called at least two more times.

## 2 LESSON 2: CONSIDER DIFFERENT PLATFORMS

One of the earliest challenges we faced was identifying which telemedicine platform best met our needs. Factors to consider included usability and accessibility, ability to have multiple participants, HIPAA compliance, privacy for providers working from home, screen sharing and visit‐tracking metrics. Our hospital system endorsed several platforms to use on a temporary basis while working to embed a platform into our electronic medical record (EMR). Many systems do not offer all desired features in one platform. For our centre, we found one system was straightforward for both providers and families, but lacked the capability to have more than two participants on the call, limiting the ability to involve trainees, ancillary providers (eg, education specialists and dieticians) or additional caregivers (eg, two parents in separate homes with a shared custody agreement). Another system allowed for multiple participants but was more cumbersome to access. Still other HIPAA‐compliant platforms required using personal accounts that may have compromised provider privacy (ie, cell phone number or email address). Other desirable features that some systems offered included screen sharing to view growth charts and device reports from insulin pumps or continuous glucose monitors (CGM), smartphone compatibility and time tracking for billing purposes.

Our hospital opted to prioritize use of the EMR‐embedded system, which operates via our patient portal and requires minimal provider training. Though simple and convenient to use, this platform does not yet include all our desired features, and it requires that families are registered for the patient portal. To accommodate different scenarios or technical issues that might arise, we promoted a flexible decision tree of back‐up web‐based platforms for providers. The decision tree was simple but effective: our preferred option was our EMR‐embedded system which operates via our patient portal. If this was unavailable (either due to the patient not having the portal account or due to technical issues), we used other commercial systems that allowed for secure video calls either via computer or smartphone. Last, if a patient lacked the technology to participate in a video visit (lack of reliable internet connection, lack of computer or phone with video capabilities) or if the technology was not functioning adequately, a phone call (audio only) was the last option. Keeping multiple options available enabled providers to move between virtual visits efficiently, taking into account both their preferences and what platform meets the needs of a given patient and family. At times, we needed to be creative with using multiple platforms simultaneously. If the Internet connection was spotty, the provider would call on the phone (requesting to be placed on speakerphone), thus allowing a continued dialogue even when the video image may have not been optimal or intermittently interrupted.

## 3 LESSON 3: FIND CREATIVE SOLUTIONS TO MEET REQUIREMENTS WHILE PROVIDING EXCEPTIONAL CARE

With changes in the way we deliver, care came changes in how we bill for and document that care. Regulations governing telemedicine procedures and billing varied by state prior to COVID‐19, and national and regional guidance has evolved rapidly during the pandemic. Initially, it was unclear whether we could continue to bill based on level of complexity, and so we had to transition to time‐based billing. To meet requirements, our corporate compliance office issued general guidelines to document a rationale for providing care via telemedicine, the platform used to conduct the visit, and, for time‐based billing, the amount of time we spent with the patient and how we utilized that time. Developing ‘virtual visit’ note types and creating new templates with required components and attestations was critical in ensuring efficient documentation that met the appropriate regulatory standards. The quick cascade of information from administration to medical teams enabled us to plan multiple steps ahead in a rapidly changing environment. Prior to the pandemic, patients were able to share insulin pump or continuous glucose monitor reports with our centre using a cloud system, but they were not routinely expected to upload data prior to their visit. Without in‐person visits, we lacked ready access to patient devices to physically download reports of blood sugars and insulin administration. To address this hurdle, our diabetes administrative team, all specialists in diabetes authorizations/downloads, contacted patients and families in the week ahead of the visits to go over the instructions on how to download their devices. Over the two days prior to the visit, the same administrative assistants would check for the downloads and, if not available, call families to remind them of the process. Once available, they would save the reports to the EMR. This process enabled providers to easily access the reports both during the virtual visit and in future encounters. For families that could not download, we asked them to write down blood glucoses from the meter and share with us (either sending a picture or reporting them to us during the visit).

It is important to note, that even with virtual visits, there were some patients that still did not show. In most cases, these were patients that had a history of not showing to the face‐to‐face visits and we were not able to reach to reschedule. In other cases, lack of connectivity was an issue and some of these families were accommodated using other platforms such as phone.

## 4 LESSON 4: CULTIVATE THE PATIENT EXPERIENCE

For patients unaccustomed to virtual visits with their provider, these encounters were met with variable feedback. Because of the acuity of the situation, no formal assessment was conducted. However, comments to providers provide some evidence of how they received this new experience. Some enjoyed the convenience of conducting the visit from home, while others lamented difficulties with technology. How to join the visit was a source of stress for some patients and families. We developed a uniform protocol for all administrative staff to follow in their communications prior to the visits, including instructions on how to access the platforms. Providers were also given guidance on setting expectations with families at the start of the visit, acknowledging the benefits and limitations of a virtual visit and normalizing their concerns. Providers were also asked to check‐in on the family's unique stressors during the pandemic, including work and education, childcare, access to medical supplies and food security. We had resources readily available, some compiled by our social workers, that were provided through the portal to families as needs were identified, including documents such as ‘Food assistance resources’, ‘Recommended list of diabetes supplies’ as well as ‘Information on how to get help to pay for medications/supplies’. If the provider felt that there was a greater need, a message was sent to the social workers through the medical record system to have them call the families. Acknowledging the significant financial hardships faced by many families as a result of COVID‐19, we attempted to be proactive in supporting our community when possible.

## 5 LESSON 5: ENGAGE FELLOWS AND ANCILLARY PROVIDERS

Many academic centres incorporate learners at all stages, including medical students, residents, fellows and APP fellows. While reducing in‐person visits and scaling up telemedicine services, it can be difficult to keep medical trainees engaged in both patient care and learning activities due to social distancing and billing requirements. We determined to continue the trainee outpatient clinical experience during COVID‐19 to maintain their education and relationships with their patients. In large part, this required exploring telemedicine platforms that allowed at least three participants in the virtual visit, such that the trainee and supervising physician could both enter the encounter. Fellows did not experience any reduction in their clinical volume (eg, number of clinics, number of patients scheduled per clinic) and additional preceptors were added when needed to accommodate 1:1 staffing for time‐based billing. In those circumstances, the fellow and preceptor would complete the entire visit together. If not completing time‐based billing, the encounters followed a similar structure to face‐to‐face visits, where the fellow would evaluate the patient, ‘step out’ of the virtual room to precept, and then the fellow and faculty members would rejoin the visit together. All other learning opportunities, including teaching conferences and case discussions, were quickly moved to a virtual platform so the whole team could participate.

In addition to trainees, certified diabetes care and education specialists, registered dieticians and social workers are integral members of the care team. Staff communicated regarding patient needs in real‐time using our EMR. Education specialists were preassigned to provider schedules; prior to the clinic, they reviewed glucose data in order to provide suggestions to the clinician. During the appointment, they were also available to address any diabetes education needs. Our dieticians created a daily ‘on call’ schedule for both annual evaluations and referrals. The on‐call dietician was able to follow‐up with the family either the same day or shortly thereafter to schedule a virtual visit. Our social work team employed similar methods to offer support or behavioural health services. This network enabled our institution to continue a multidisciplinary approach to care undisturbed.

## 6 LESSON 6: IDENTIFY REMAINING BARRIERS

Despite our success, there are remaining challenges that are likely to be common among institutions implementing telemedicine. In some cases, poor cell phone or Internet service affected the connection leading to delays or interruptions; in others, patients lacked access to a smartphone, tablet or computer that would enable them to complete video visits. If this was the case, we did offer telephone visits to ensure continued access to care for all our patients (<10% of the visits). An additional technological concern is translation services. Pittsburgh is fairly homogenous, and the great majority of the families speak English. However, our institution has a built‐in system for remote translation via telephone if there is no provider within our practice who speaks the native language.

Administrative challenges include a shift in daily responsibilities. To increase portal registration, we worked with the information technology group to simplify the sign‐in process and make available simple instructions by phone or online. We offered alternative solutions to continue visits for those without portal access and advised families how to download their devices at home. The added workload was accomplished by diverting efforts from other administrative duties that we did not have as face‐to‐face visits were not occurring. Added resources may be needed to sustain this type of effort indefinitely.

Not all aspects of an in‐person visit translate well to telemedicine evaluations. Virtual visits limit the provider's ability to do a comprehensive examination, which is needed to assess for growth, pubertal status, lipohypertrophy and physical findings of associated conditions (eg, autoimmune thyroid disease or adrenal insufficiency). One meaningful intervention has been to have medical assistants contact families ahead of time to obtain an accurate weight and height at home, though this does not replace a physical exam.

Similarly, another challenge is obtaining necessary laboratory studies. For those patients with CGMs, we were able to use key CGM metrics to evaluate glycaemic control. In certain situations (eg, no CGM, due for annual screening, needed for management decision, family request), a script was sent through the portal or by mail in order to get laboratory tests locally. Lastly, for our patients without diabetes devices or incapable of downloading at home, we provided advance notice to the family to write down and submit written records for our review.

Lastly, institutions must broadly consider the future of telemedicine services in the context of state law and billing requirements. For providers who serve patients from multiple states, licensure for telemedicine across state lines need to be considered, similar to face‐to‐face visits. There will likely be different approaches to how states use telemedicine and allow for out of state usage. A remaining question surrounds provider reimbursement for telemedicine after the pandemic. We found that it was very important to work closely with our institutional managed care/contracting department to best understand our local dynamics. Having a good understanding of state Medicaid's current approach to telemedicine is essential, and keeping up to date on national guidance to anticipate future changes as well.

All providers should argue for continued coverage. In the coming months, legislative policy will be instrumental in sustaining this new model of care. There are unquestionably opportunities in streamlining telemedicine for clinical care and determining which services can be best delivered via a telehealth model. More research is needed on effectiveness and the appropriate way to implement it in a long‐term care model, but telemedicine offers opportunities to reach patients who are otherwise limited in obtaining care because of different reasons such as geography, health of patients/caregivers, etc. A critical component will be formalizing systems for obtaining patient feedback on their experiences with telemedicine, allowing for ongoing quality improvement.

## 7 LESSON 7: STAY POSITIVE

We cannot overstate the importance of maintaining a positive attitude through this very worrisome time. Weekly virtual meetings, email updates and identifying a point person from each stakeholder group contributed to the division's co‐operative can‐do attitude. We are taking a moment to reflect on the many ways in which this experience will influence patient care for the better, even after the restrictions necessitated by this pandemic are lifted. We have significantly increased patients' registration for and comfort in using the patient portal, which is the preferred method for communication with the healthcare team for laboratory test results, prescription refills and non‐urgent questions. Patients and families are learning how to upload information from their insulin pumps and continuous glucose monitors from home, which will both streamline future in‐person visits and allow for review of the data by the diabetes team in between visits as needed. Additionally, increased uptake in telemedicine usage may enhance access to regular, routine care for patients when distance, weather or other circumstances which might prohibit their travelling to our facility. Beyond clinical care, new opportunities exist for research to explore the effectiveness of this modality for other target populations, such as emerging adults as part of transition care services.

In summary, the COVID‐19 pandemic has generated incredible challenges around the world. Healthcare systems have needed to care for an influx of ill patients while still attempting to deliver care for patients with chronic medical conditions. Telemedicine has become a new standard of care. Despite hurdles and some remaining challenges, the infrastructure developed for telemedicine on a large scale will leave a lasting imprint on many diabetes centres. Moving forward, the experience we have gained will continue to drive innovation in developing and evaluating new strategies to optimize care for children and adolescents with diabetes.

## CONFLICT OF INTEREST

The authors are all employed by UPMC Children's Hospital of Pittsburgh. They have no significant financial conflicts of interest to disclose.

## AUTHOR CONTRIBUTIONS

All authors participated in the telemedicine committee, and IL, RM, CM and AF conceptualized this manuscript. CM and AF drafted the manuscript. ER contributed the clinical data, which IL reviewed, summarized and assimilated into table format. All authors reviewed, edited and agreed upon the final version of the manuscript.

## Data Availability

The data that support the findings of this study are available from the corresponding author upon reasonable request.
